# Healthcare system preparedness as a mediating factor in climate-driven tuberculosis outcomes: an ecological study

**DOI:** 10.1186/s12913-026-14777-8

**Published:** 2026-05-22

**Authors:** Wahyu Septiono, Fadhaa Aditya Kautsar Murti, Ibnu Muyassar, Popy Yuniar, Indang Trihandini, Amalia Hasnida, Zenlin Kwee

**Affiliations:** 1 Department of Biostatistics and Population Studies, Faculty of Public Health, University of Indonesia, Kampus Baru UI, A Building 2nd Floor, Depok, West Java 16424 Indonesia; 2 Social Determinants of Health Center for Equity, Faculty of Public Health, University of Indonesia, Depok, West Java Indonesia; 3 Erasmus School of Health Policy and Management, Erasmus University Rotterdam, Rotterdam, The Netherlands; 4 Department of Values, Technology and Innovation, Faculty of Technology, Policy and Management, Delft University of Technology, Delft, The Netherlands

**Keywords:** Tuberculosis, Health system, Healthcare preparedness, Climate change, Mediation analysis

## Abstract

**Background:**

Climate change may influence tuberculosis (TB) transmission through environmental and health system pathways. It is important to understand how the health system can adapt to reduce the impact of climate change on TB transmission.

**Methods:**

We conducted an ecological study using district-level data from the Indonesian Health Facility Research Survey, the National Health Insurance data sample registry, and monthly climate indicators (temperature, humidity, precipitation) for 2019. Mediation analysis was applied to assess the role of healthcare preparedness in mediating the relationship between climatic variability and TB incidence.

**Results:**

Healthcare preparedness fully mediated the association between humidity and TB incidence. The indirect effect through healthcare preparedness was statistically significant (*α × β* = 0.0021, 95% CI: 0.0008, 0.0035), while the direct effect was not (*c*^*1*^ = 0.0102, 95% CI: -0.0013, 0.0217). Approximately 17.1% of the total effect of humidity on TB (*c* = 0.0123, 95% CI: 0.0009, 0.0237) mediated through healthcare preparedness. For temperature, the relationship with TB was partially mediated, with a small but significant negative indirect effect (*α × β* = -0.0002, 95% CI: -0.0003, -0.0001), indicating that adequate healthcare may offset about 14.3% of temperature-related TB risk, while a significant direct effect remained. No significant mediation was found for precipitation.

**Conclusion:**

These findings highlight the importance of targeted investments to strengthen healthcare systems in high-burden, climate-vulnerable districts. Integrating climate adaptation into TB control programs and enhancing surveillance are essential to ensure accurate burden estimation, effective resource allocation, and resilience against the combined challenges of climate variability and TB transmission.

**Supplementary Information:**

The online version contains supplementary material available at 10.1186/s12913-026-14777-8.

## Introduction

Tuberculosis (TB) remains a major global public health issue, with an estimated 10.8 million cases and 1.25 million deaths reported in 2023 [1, 2]. The burden falls disproportionately in low- and middle-income countries (LMICs), which account for nearly two-thirds of global cases [3]. In many LMICs, limited health system capacity, including inadequate diagnostic infrastructure and weak surveillance systems, can substantially hinder the implementation and effectiveness of TB eradication programs [4]. Moreover, climatic factors such as rising temperatures and humidity may intensify the TB burden, particularly in settings with limited adaptive capacity [5–7]. Climate variability can influence TB transmission through both direct and indirect pathways. Changes in temperature and humidity can affect the environmental persistence of *Mycobacterium tuberculosis*, shaping how long the bacteria remain viable and infectious in airborne droplets [8]. In addition, climate-related stressors may disrupt healthcare service delivery and access, thereby affecting timely diagnosis, treatment adherence, and overall TB program performance [8, 9]. Collectively, limitations in health system capacity and environmental pressures hinder effective disease control and pose challenges to TB elimination in resource-limited settings.

Empirical evidence indicates that climatic factors such as higher temperatures, increased precipitation, and rising humidity play an important role in shaping TB incidence and transmission dynamics [10, 11]. Liyew et al. (2024) reported a positive association between high humidity and TB transmission, suggesting that moist conditions may prolong *Mycobacterium tuberculosis* survival and facilitate airborne spread [11]. Extreme climatic conditions may influence TB transmission by affecting the environmental persistence of the pathogen. High humidity can prolong the stability of droplet nuclei, enabling the bacteria to remain viable and airborne for extended periods, whereas temperature variations may alter aerosol dynamics and bacterial survival [8, 11]. Moreover, climate extremes may indirectly increase transmission by shaping indoor environments, including ventilation patterns and population crowding [8]. Such dynamics are particularly pronounced in tropical and subtropical regions, where both climatic extremes and health system weakness are more prevalent [12].

Health system preparedness has become a critical global concern in the context of increasing climate variability and the rising threat of future infectious disease outbreaks. Essentially, health system preparedness refers to the ability to anticipate, withstand, adapt to, and respond effectively to shocks such as epidemics or environmental changes while maintaining essential functions and service delivery [13]. For example, the COVID-19 pandemic exposed weakness across health systems worldwide, showing how fragile infrastructures and limited preparedness can quickly lead to overwhelmed services during crisis [14, 15]. In the context of TB, health system preparedness includes the capacity to ensure timely case detection, accurate diagnosis, uninterrupted treatment, and effective surveillance and coordination, particularly under environmental and system-related stressors such as climate variability. The resilience and operational strength of a health system can either buffer against or intensify the adverse impacts of climate variability on TB outcomes. From a conceptual perspective, health system preparedness may act as a mediator linking climate variability to TB outcomes [8, 9], whereby differences in system readiness influence the extent to which environmental stressors affect disease detection, management, and transmission dynamics. Despite growing evidence linking climate factors to TB incidence, relatively little empirical work has explored how health system preparedness shapes this relationship. The mediating role of system capacity, whether in mitigating risks or amplifying vulnerabilities, remains underexamined. This conceptual and empirical gap limits the development of adaptive, context-specific interventions that could protect vulnerable populations from the compounded effects of climate change and TB.

Indonesia has not yet implemented a specific strategy on health system preparedness that explicitly integrates climate change into TB eradication efforts while evidence on the resilience of Indonesia’s healthcare system remains mixed. A cross-sectional study conducted in Depok City reported an average resilience score of 61.7% for community health centers, with strong capacity in workforce resources (79.3%) but notably lower scores in financial preparedness (42.3%) [16]. The COVID-19 pandemic in Indonesia also placed considerable strain on health services, with stress and burnout negatively affecting performance and overall responsiveness [17]. Therefore, although these findings indicate that despite progress, Indonesia’s healthcare system remains vulnerable to external shocks. Particularly, the TB incidence raises concern, as TB case notifications in Indonesia showed a sharp decline from 205 per 100,000 population in 2019 to 140–154 per 100,000 in 2020–2021, likely due to COVID-19 disruptions, before rebounding significantly to 254 in 2022 and 286 in 2023 as TB services recovered [2]. In 2024, WHO reported that Indonesia remains one of the eight countries contributing to about two-thirds of the global TB burden [2].

This study assesses the mediating role of healthcare system preparedness across Indonesian districts in the relationship between climate variability and TB incidence at the district level. To our best knowledge, this study provides the first empirical test of health system preparedness as a mediating mechanism between climate variability and TB outcomes, using multiple national datasets from Indonesia (i.e., the national health insurance registry, data from nationwide health facility surveys, and district-level climate indicators). An ecological study design was employed, with the district serving as the unit of analysis, enabling the assessment of the district’s population-level associations while accounting for variations in health system capacity across districts.

## Methods

### Data source

This study integrated three primary data sources, consisting of: [1] national TB case records from Indonesia’s national health insurance [2], district-level climate data, and [3] health system preparedness data from a national facility survey. TB case data were obtained from *Jaminan Kesehatan Nasional* (JKN), Indonesia’s national health insurance program managed by the *Badan Penyelenggara Jaminan Sosial Kesehatan* (BPJS Kesehatan). The administrative database includes individual-level records on diagnoses, referrals, clinical procedures and healthcare expenditures. As of January 31, 2023, the program covered approximately 249 million people, representing nearly 90% of the Indonesian population [18]. Given that the data were derived from claims records, we aggregated multiple claims per individual to classify individuals as having TB or not. For this study, we used a sample of registry data in 2019, comprising a total of 573,610 individuals.

The climatic dataset was derived from combination observational data from ground-based monitoring stations managed by Indonesia’s Meteorology, Climatology, and Geophysical Agency (Badan Meteorologi, Klimatologi, dan Geofisika, BMKG) and reanalysis data from ERA5 developed by the European Centre for Medium-Range Weather Forecasts (ECMWF). Precipitation (mm) data were obtained from BMKG, which applies a statistical downscaling technique integrating reanalysis models, ground station observations, and satellite-derived estimates. From ERA5, key meteorological variables were extracted, including 2-meter air temperature (°C) and dew-point temperature (°C). Relative humidity (%) was estimated using an empirical method based on the relationship between air temperature and dew-point temperature, given the lack of direct measurement. ERA5 provides gridded climate estimates generated through data assimilation, combining observational data (e.g., satellites and weather stations) with a numerical weather prediction model to produce spatially continuous atmospheric fields. These gridded data were spatially aggregated to the district level by averaging all grid cells within each district boundary. All variables were aggregated to reflect monthly averages.

We employed data from the 2019 Riset Fasilitas Kesehatan (RISFASKES), the second round of the nationally representative facility-based health survey following the 2011 survey, conducted by the Ministry of Health as a cross-sectional assessment rather than monthly observations. The survey aims to evaluate the availability, readiness, and performance quality of healthcare service delivery across multiple levels of the health system, including primary health centers (puskesmas), hospitals, clinics, and other healthcare providers. The survey assessed the availability of infrastructure and equipment, adequacy of human resources, service delivery capacity, functionality of health information systems, availability of essential drugs and medical supplies, and the effectiveness of referral mechanisms [19], which correspond to key components outlined in the WHO’s Service Availability and Readiness Assessment (SARA) framework. RISFASKES 2019 used a total coverage approach for public health offices (514 district/city health offices) and puskesmas (9,909 facilities), while hospitals (144), clinics, and other providers were selected through stratified sampling [19].

### Measurement

#### TB cases

This study focused exclusively on identifying cases of all-form TB both hospitalization and non-hospitalization. To detect these cases within the dataset, we used diagnostic codes based on the International Classification of Diseases, 10th Revision (ICD-10), specifically codes A15, A17, A18, and A19, which correspond to TB [20]. We then calculated the monthly proportion of individuals diagnosed with TB across all districts from January to December 2019.

#### Climate indicators

The variable used to operationalize monthly climate indicators was the mean values of temperature, precipitation, and relative humidity observed at the district level between January and December 2019. Temperature, expressed in degrees Celsius (°C), served as a measure of the thermal environment within each district. Precipitation, quantified in millimeters (mm), captured the cumulative volume of rainfall over a specified period. Relative humidity, reported as a percentage (%), reflected the proportion of water vapor in the air, influencing local atmospheric conditions.

#### Healthcare preparedness

To assess the preparedness level of healthcare services, we utilized the WHO Standards and Benchmarks for TB Surveillance and Vital Registration Systems (2014) [21] in combination with the strategies proposed by Hopewell et al. (2006) [22]. This combination was intended to capture both system-level surveillance capacity (WHO framework) and operational components of TB control at the service delivery level (Hopewell et al.), thereby providing a more comprehensive conceptualization of healthcare preparedness for TB control. From these frameworks, domains were selected based on their operational availability within RISFASKES 2019 dataset. Specifically, we included domains that [1] directly relate to TB diagnosis, treatment, and surveillance capacity [2], are consistently measured across facilities in RISFASKES, and [3] reflect modifiable components of health system readiness. The selected domains included health workforce, diagnostic capacity, infrastructure and logistics, information systems, supportive services, and governance and coordination.

The health workforce domain was measured by the availability of staff certified in DOTS (Directly Observed Treatment, Short-course). Diagnostic capacity encompassed the availability of laboratories dedicated to sputum microscopy, molecular testing, and Rontgen, as well as established procedures for TB management, and drug-resistant TB cases management for TB-HIV coinfection. Infrastructure and logistics were defined by the presence of access to first- and second-line TB medications, and the availability of isolation rooms for infectious TB patients. The information system domain was evaluated based on the presence of a TB case registration platform integrated with the national surveillance system. Finally, governance and coordination were defined by the availability of Standard Operating Procedures (SOPs).

Each variable within all assessed domains was dichotomously scored. A value of 1 was assigned if the component was present, and 0 if it was absent. The overall score was derived by summing up the values of all assessed variables, resulting in a range of 0 to 12 points. The median value was employed as the cut-off to classify the level of healthcare resilience, with scores below the median categorized as low resilience and scores equal to or above the median categorized as high resilience. The proportion of resilient healthcare facilities at the district level was then calculated as the percentage of facilities classified as highly resilient out of the total number of assessed healthcare facilities.

### Data analysis

**Fig. 1:**
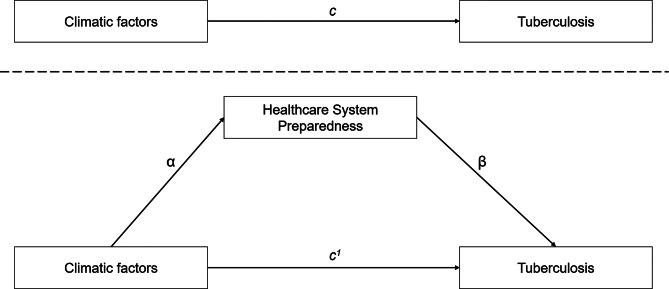
Conceptual framework of the mediation analysis examining the role of healthcare system preparedness in the association between climatic factors and tuberculosis

The data analysis was conducted in descriptive and mediation analyses stages using aggregated data at the district level as the unit of analysis, encompassing 514 districts. The descriptive analysis explored monthly trends in key climatic variables and TB prevalence from 2019. Spatial distributions of the proportion of health facilities with adequate TB preparedness and age-standardized TB prevalence were mapped across 514 districts using QGIS 3.34 (QGIS Development Team, 2023; http://qgis.org). A conceptual framework illustrating the hypothesized mediation model is presented in Fig. 1. This model depicts the assumed pathways through which climatic variables (temperature, precipitation, and humidity) influence TB prevalence, both directly and indirectly through health system preparedness as a mediating factor. For the mediation analysis, a series of linear regression models were constructed to examine the potential mediating role of healthcare preparedness, indicated from direct (*c*^*1*^), indirect (*α × β*), and total effects (*c = (α × β) + c*^*1*^*).* Regression coefficients mediation analysis and their corresponding 95% confidence intervals (95% CI) were utilized to estimate the associations. To improve interpretability and numerical stability of the regression estimates, climatic variables were rescaled (temperature per 5 °C, precipitation per 1,000 mm, and humidity per 100 units) prior to analysis. For the supplementary analyses (Supplementary Tables 1–3), we conducted sensitivity analyses by restricting the sample to capital cities, districts with low TB prevalence, and districts with high TB prevalence. STATA 17 (StataCorp LLC, College Station, TX, USA) was utilized to perform mediation analysis under SEM (structural equation modeling).

## Results

**Fig. 2:**
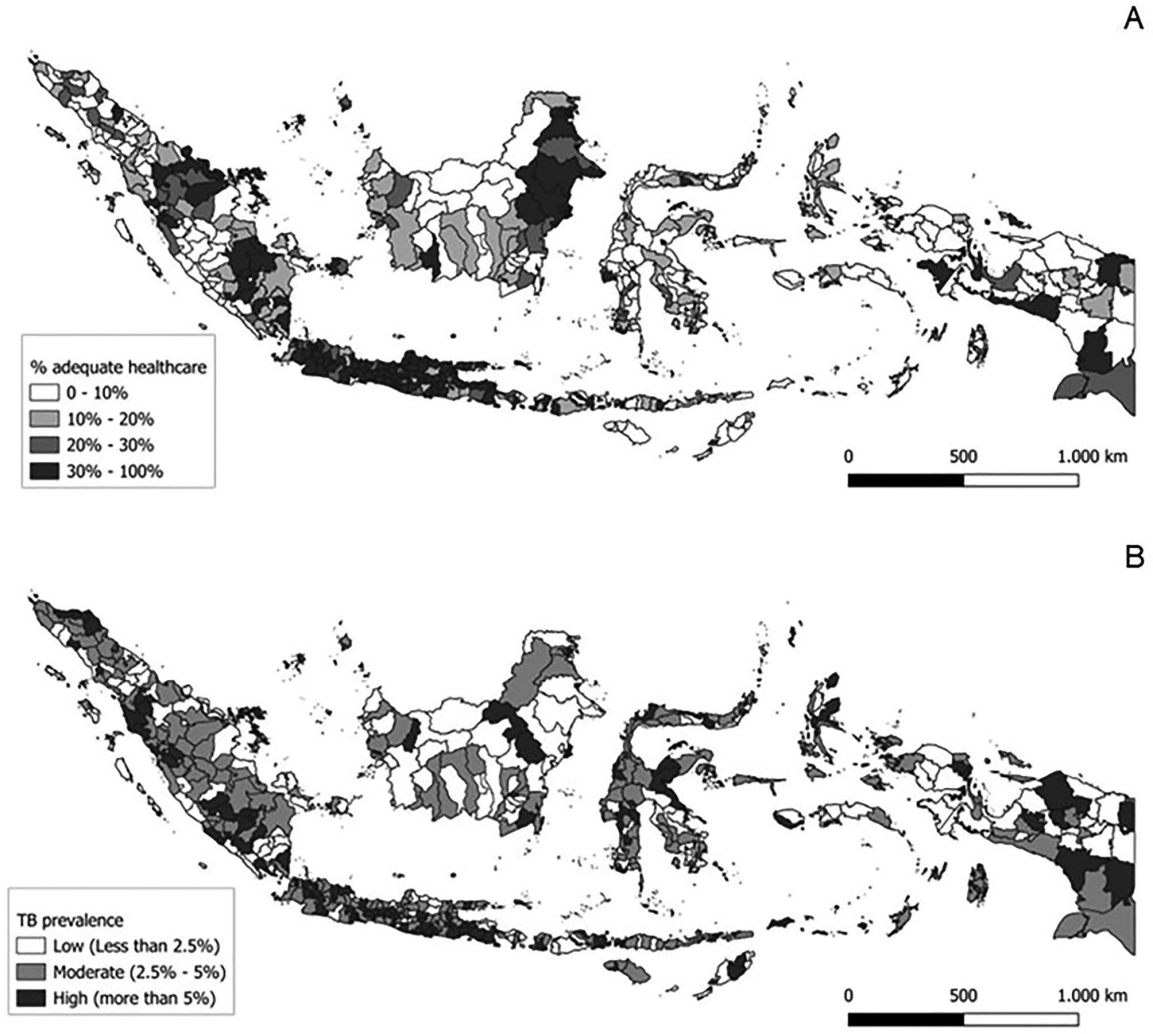
Spatial analysis of district-level age-standardized tuberculosis prevalence and the proportion of health facilities with adequate preparedness (*N* = 514)

Figure 2 presents the proportion of health facilities with adequate preparedness (Panel A) and the spatial distribution of district-level age-standardized TB prevalence (Panel B) across Indonesia. Panel A reveals geographic disparities in healthcare preparedness, with many districts in eastern Indonesia, particularly in Papua, Nusa Tenggara, Maluku, and Sulawesi, as well as parts of Sumatra and Kalimantan, exhibiting the lowest levels of adequately prepared health facilities (0–10%). In contrast, higher levels of preparedness (30–100%) are more commonly observed in districts across Java, eastern Kalimantan, and central to southern Sumatra. The spatial distribution indicates that districts with both high and low levels of TB preparedness tend to form clusters, suggesting that neighbouring districts may often share similar levels of healthcare readiness. Panel B exhibits the spatial distribution of TB prevalence, highlighting clusters of high prevalence (above 5%) predominantly in districts across Java. Elevated TB prevalence is also observed in several areas of Sumatra, Sulawesi, and Papua.

**Fig. 3:**
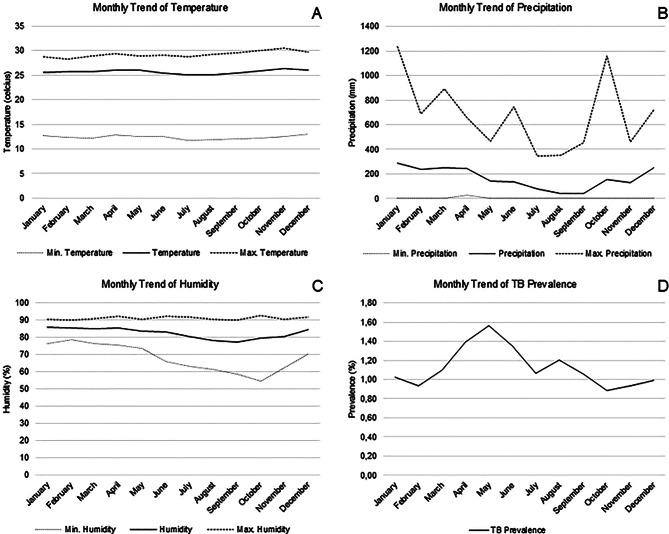
Monthly average trend of temperature (**A**), precipitation (**B**), relative humidity (**C**), and age-standardized TB prevalence (**D**) in Indonesia in 2019

Figure 3 illustrates the monthly average trends of temperature (A), precipitation (B), relative humidity (C), and age-standardized TB prevalence (D) in Indonesia during 2019. Panel A shows relatively stable temperature patterns throughout the year, with average values ranging between 26 °C and 28 °C, and minimal seasonal fluctuation in both minimum and maximum temperatures. Panel B reveals marked variability in precipitation, with higher rainfall observed in the early months (January–March), a decline during the mid-year dry season (June–September), and a rising precipitation in the end of the year (September-December). Panel C indicates that relative humidity followed a similar seasonal trend, gradually decreasing from February to October, reaching its lowest in October, before increasing again in the final months of the year. Panel D presents the monthly trend of age-standardized TB prevalence, which peaked in May and declined gradually in the following months.

Table 1 shows the results of the mediation effect estimates of healthcare preparedness in the associations between climatic factors and TB. Improved healthcare preparedness for TB was associated with the reduced TB prevalence at the district level (*c* = -0.0042, 95%CI: -0.0068, -0.0017). An increase of 5 degrees in temperature was significantly associated with higher TB prevalence (c = 0.0016, 95%CI: 0.0003–0.0028), while the associations with precipitation and humidity were positive but not statistically significant. The results demonstrate a pattern consistent with full mediation in the pathway linking humidity and TB incidence, as the indirect effect through healthcare preparedness was statistically significant (α × β = 0.0021, 95%CI: 0.0008, 0.0035), while the direct effect of humidity on TB was not significant (*c*^*1*^ = 0.0102, 95%CI: -0.0013, 0.0217). Approximately 17.1% of the total effect of humidity on TB was mediated by healthcare preparedness, as reflected in the total effect (*c* = 0.0123, 95%CI: 0.0009, 0.0237). For temperature and TB, the association was partially mediated by healthcare preparedness, with a small but significant negative indirect effect (α × β = -0.0002, 95%CI: -0.0003, -0.0001), suggesting that more adequate healthcare may offset around 14.3% of temperature-related TB risk. Sensitivity analyses (Supplementary Tables 1–3) show that the mediation effect varies by context, being stronger in high-prevalence districts and weaker in low-prevalence areas and capital cities.

**Table 1:** Mediation of the associations between healthcare preparedness and tuberculosis: regression coefficients and 95% confidence intervals for the indirect effect (i.e. α × β)

		Mean (SD)	Direct effect (c^1^)	95% CI	Indirect effect (α × β)	95% CI	Total effect (c)	95% CI
Temperature (per 5 °C)		5.14 (0.43)	0.0016	0.0003, 0.0028	-0.0002	-0.0003, -0.0001	0.0014	0.0001, 0.0026
Precipitation (per 1,000 mm)		0.17 (0.14)	0.0009	-0.0036, 0.0054	-0.0002	-0.0005, 0.0001	0.0007	-0.0039, 0.0052
Humidity (per 100)		0.82 (0.06)	0.0102	-0.0013, 0.0217	0.0021	0.0008, 0.0035	0.0123	0.0009, 0.0237
***Potential mediator***								
Adequate healthcare (%)		0.94 (2.01)	-0.0042	-0.0068, -0.0017			-0.0042	-0.0068, -0.0017

## Discussion

### Key findings

Distinct seasonal trends in climate variables and TB prevalence were observed across Indonesia in 2019. TB prevalence peaked in May, following a period of relative humidity. Spatial analysis revealed a potential of geographic clustering, with high TB prevalence and low healthcare preparedness co-occurring in several districts, particularly in eastern Indonesia and parts of Sumatra, Sulawesi, and Kalimantan. Furthermore, healthcare preparedness fully mediated the association between humidity and TB incidence, with 17.1% of the effect transmitted through this pathway. In contrast, the association between temperature and TB was partially mediated, with healthcare preparedness mitigating approximately 14.3% of the temperature-related TB burden. These findings highlight the critical role of district-level healthcare capacity in mediating the impact of climatic factors on TB outcomes.

### Potential limitations

Several limitations should be considered when interpreting the results. First, the JKN data sample registry does not contain detailed information on individual health behaviors and lifestyle factors, which limits the ability to control for behavioral confounders in examining the associations between climatic factors, healthcare system preparedness, and TB. Second, as the data were derived from health insurance claims, TB cases among individuals who were not enrolled in JKN or who did not seek formal healthcare services may not be captured, potentially leading to an underestimation of the actual disease burden. Third, climatic exposures were assessed at aggregated geographic levels and may not fully reflect individual-level exposure, particularly in areas with substantial microclimatic variation. Fourth, to the best of our knowledge, there is currently no validated instrument available to quantify preparedness for this issue. As a result, the scoring approach used in this study may lead to potential overestimation or underestimation across domains. Future research should focus on developing and validating a standardized instrument to more accurately assess preparedness levels. Fifth, the use of survey-based healthcare preparedness data may not capture meaningful month-to-month variation, and the study design, together with the use of aggregated ecological data at the monthly level, may not fully reflect underlying variability and may attenuate observed associations; furthermore, unmeasured contextual factors (e.g., behavioural, socioeconomic, and health system heterogeneity) may partly explain the relatively modest effect sizes.

### Interpretation of results

This study indicates that healthcare preparedness fully mediates the relationship between humidity and TB incidence, suggesting that humidity influences TB outcomes primarily through health system pathways rather than direct effects alone. Elevated humidity may contribute to TB transmission through several mechanisms. Biologically, humid conditions may prolong the survival of *Mycobacterium tuberculosis* and increase susceptibility to respiratory infections [23]. Environmentally, high humidity is often associated with reduced ventilation and increased indoor crowding, which can facilitate transmission [24]. Behaviorally, humid and adverse weather conditions may alter mobility patterns and delay healthcare-seeking behavior, potentially leading to delayed diagnosis and treatment [25]. At the health system level, high humidity may disrupt service delivery, outreach activities, and logistics, thereby affecting surveillance, early case detection, and treatment continuity [5–7]. Districts with stronger healthcare preparedness may be better equipped to mitigate these risks through robust surveillance systems, improved laboratory capacity, public education, and timely treatment. Li et al. (2014) found that increased TB investments in China, especially in laboratory capacity and healthcare workforce, significantly reduced TB transmission despite high transmission during high humidity period due to improved mitigation [26]. In this context, healthcare preparedness is particularly important in ecological research, as system-level characteristics often determine how climate exposure leads to disease consequences [27]. The results show how important it is to make district-level health systems stronger so that those can protect people from the consequences of climate variability, especially in high-humidity regions.

In contrast, healthcare preparedness was found to partially mediate the relationship between temperature and TB incidence. The significant direct effect of temperature on TB suggests that higher temperatures may independently contribute to increased transmission through both biological and behavioral pathways. Elevated temperatures can impair immune function, increase the reactivation of latent TB [28], and lead to behavioral adaptations such as increased indoor crowding [29], all of which can elevate TB risk. While healthcare preparedness plays a critical role in mitigating part of this impact, it does not fully eliminate the temperature-related risk. This partial mediation highlights that temperature influences TB incidence through both health system–dependent and independent mechanisms. Therefore, while investments in healthcare infrastructure are vital, broader multisectoral climate adaptation strategies are also needed to reduce TB vulnerability in the context of rising temperatures.

This study observed a potential spatial clustering of TB prevalence and adequate preparedness of healthcare, suggesting the possibility of localized or inter-district transmission dynamics. This could potentially be driven by population mobility between adjacent districts [30, 31]. Similar patterns have been observed in Ethiopia, showing that TB transmission can cluster along geographic or administrative boundaries, highlighting the importance of considering regional connectivity in TB control strategies [32]. The identification of TB prevalence clusters underscores the need for coordinated area-based interventions that extend beyond administrative borders, adopting a horizontal diffusion approach that enables collaborative action among adjacent districts. However, some minor clusters were observed in districts with both low TB prevalence and weaker healthcare preparedness. Underreporting in these areas may mask the actual disease burden, leading to potential misinterpretation of spatial patterns. These areas may reflect underreporting due to weak surveillance systems rather than an actual absence of TB burden. Strengthening TB surveillance in these regions is critical to improve case detection, ensure accurate burden estimation, and avoid misinformed resource allocation.

### Conclusion

This study demonstrates that climatic factors, particularly temperature and humidity, influence TB incidence in Indonesia largely through district-level healthcare system preparedness. While the effect of humidity on TB was fully mediated by healthcare capacity, temperature showed both mediated and direct effects, indicating multiplate pathways linking climate and TB risk. The spatial overlap of high TB burden and low healthcare preparedness highlights persistent geographic inequities. In climate-sensitive districts, targeted interventions such as expanding diagnostic capacity, strengthening community-based case detection, and ensuring continuity of treatment services should be prioritized to mitigate climate-related disruptions in TB control. Resource allocation for TB programs should be guided not only by disease burden but also by climatic vulnerability profiles, prioritizing areas exposed to persistently high humidity and rising temperatures. Moreover, aligning national TB elimination efforts with broader climate resilience and health system preparedness agendas can help future-proof TB programs against the growing health impacts of climate variability. These findings support a shift from solely case-based TB interventions toward integrated, climate-informed, and system-strengthening approaches to achieve sustainable TB control and elimination.

## Supplementary Information

Below is the link to the electronic supplementary material.

Supplementary Material 1

## Data Availability

The data utilized in this study were obtained from the JKN administered by BPJS Kesehatan. Access to these datasets is available upon request to BPJS Kesehatan. The authors do not have permission to share data.

## Abbreviations

BMKG: Badan meteorologi, klimatologi, dan geofisika
BPJS: Badan penyelenggara jaminan sosial
DOTS: Directly observed treatment, short-course
ECMWF: European centre for medium-range weather forecasts
ICD: International classification of diseases
JKN: Jaminan kesehatn nasional
LMIC: Low- and middle income country
RISFASKES: Riset fasilitas kesehatan
SARA: Service availability and readiness assessmen
SEM: Structural equation modeling
SOP: Standard operating procedures
TB: Tuberculosis
WHO: World Health Organization
