# Supplementary material for: Healthcare system preparedness as a mediating factor in climate-driven tuberculosis outcomes: an ecological study
Source: BMC Health Serv Res. 2026 May 22;26:998. doi: 10.1186/s12913-026-14777-8 (PMC13383538; doi:10.1186/s12913-026-14777-8)
Supplement: Supplementary file 1 — Supplementary Material 1 [file 12913_2026_14777_MOESM1_ESM.pdf]

Supplementary Table 1. Sensitivity analysis using capital cities only

	**Direct effect (c^1)**	95% CI	**Indirect effect (α×β)**	95% CI	**Total effect (c)**	95% CI
Temperature (per 5°C)	0.0005	-0.0039, 0.0049	0.0005	-0.0002, -0.0013	0.0001	-0.0001, 0.0002
Precipitation (per 1,000mm)	-0.0037	-0.0143, 0.0069	-0.0007	-0.0018, 0.0005	0.0010	-0.0034, 0.0054
Humidity (per 100)	0.0237	-0.0034, 0.0508	0.0021	-0.0013, 0.0055	-0.0044	-0.0150, 0.0062
Potential mediator						
Adequate healthcare (%)	0.0001	-0.0068, -0.0017			0.0258	-0.0013, 0.0527

Supplementary Table 2. Sensitivity analysis using districts with low TB prevalence only

	**Direct effect (c^1)**	95% CI	**Indirect effect (α×β)**	95% CI	**Total effect (c)**	95% CI
Temperature (per 5°C)	0.0002	-0.0001, 0.0005	0.0002	0.0001, 0.0003	0.0004	0.0001, 0.0006
Precipitation (per 1,000mm)	0.0004	-0.0006, 0.0005	0.0002	0.0001, 0.0003	0.0006	-0.0004, 0.0015
Humidity (per 100)	-0.0008	-0.0006, 0.0013	-0.0013	-0.0017, -0.0009	-0.0021	-0.0044, 0.0003
Potential mediator						
Adequate healthcare (%)	0.0002	0.0001, 0.0003			0.0002	0.0001, 0.0003

Supplementary Table 3. Sensitivity analysis using districts with high TB prevalence only

	**Direct effect (c^1)**	95% CI	**Indirect effect (α×β)**	95% CI	**Total effect (c)**	95% CI
Temperature (per 5°C)	-0.0020	-0.0052, 0.0012	-0.0005	-0.0013, 0.0002	-0.0025	-0.0058, 0.0008
Precipitation (per 1,000mm)	-0.0093	-0.0019, 0.0006	-0.0005	-0.0028, 0.0017	-0.0099	-0.0200, 0.0003
Humidity (per 100)	0.0049	0.0233, 0.0751	0.0155	0.0090, 0.0220	0.0647	0.0383, 0.0911
Potential mediator						
Adequate healthcare (%)	-0.0003	-0.0004, -0.0001	0.0049	0.0233, 0.0751	-0.0003	-0.0004, -0.0001
